# For Sensitive Dentine

**Published:** 1874-12

**Authors:** 


### FOR SENSITIVE DENTINE.

For about two months we have been using, to relieve or mitigate sensitive dentine during operations of filling, a preparation sent us by Dr. Allport, with the request "try it." It is a nostrum, which the inventor and maker denominates, "Fluid Lightning" from the instantaneous relief it affords in many cases of headache, neuralgia etc. Dr. Allport, some months ago was induced to try it in a very severe and aggravated case of sensitive dentine which he had in charge. In that case he found such immediate and effective relief, as to induce him to continue his experiments, and to his great gratification he has found it to be the most effectual obtunder of sensitive dentine in use. Our experiments correspond with those of Dr. A., and from the success thus far we feel that we shall hardly have occasion to seek for or use anything else. It is instantaneous in its action and we have found no case since beginning its use, that could not be easily controlled and operated without much discomfort to the patient. Others have been testing it with about the same results. It is prepared by Dr. J. S. Cram of Chicago. Arrangements will be made by which it can be obtained at the dental depots.

Some more appropriate name will probably be given to it, at least to that put up for dental use. Patients usually imagine there is Lightning enouugh in the ordinary operations upon the teeth without any extra touches.

